# Real-life diagnostic performance of opportunistic computed tomography screening for osteoporosis in Brazilian adults: a retrospective validation study

**DOI:** 10.1590/0100-3984.2025.0073

**Published:** 2025-12-15

**Authors:** Fabio Brandão Yoshimura, Írline Cordeiro de Macedo Pontes, Erina Megumi Nagaya Fukamizu, Eduardo Kaiser Ururahy Fonseca, Patricia Yokoo, Akemi Osawa, Adham do Amaral e Castro

**Affiliations:** 1 Hospital Israelita Albert Einstein, São Paulo, SP, Brazil; 2 Instituto do Coração do Hospital das Clínicas da Faculdade de Medicina da Universidade de São Paulo (InCor/HC-FMUSP), São Paulo, SP, Brazil; 3 Departmento de Diagnóstico por Imagem, Escola Paulista de Medicina da Universidade Federal de São Paulo (EPM-Unifesp), São Paulo, SP, Brazil

**Keywords:** Bone density, Osteoporosis/diagnostic imaging, Prevalence, Absorptiometry, photon, Tomography, X-ray computed., Densidade óssea, Osteoporose/diagnóstico por imagem, Prevalência, Absorciometria de fóton, Tomografia computadorizada.

## Abstract

**Objective:**

To evaluate the diagnostic accuracy of computed tomography (CT) for detecting osteoporosis in Brazilian adults, using dual-energy X-ray absorptiometry (DXA) as the reference standard.

**Materials and Methods:**

We conducted a retrospective analysis of adults over 50 years of age who underwent CT (chest, abdominal, or lumbar scans) and DXA within a 60-day interval, between January 2012 and December 2022. Vertebral bone attenuation at L1 was quantified in Hounsfield units. Thresholds of > 160 HU and < 100 HU were used in order to classify bone as normal and osteoporotic, respectively. Two musculoskeletal radiologists, working independently, performed subjective classifications. Sensitivity, specificity, predictive values, and interobserver agreement (Cohen’s kappa) were calculated. The influence of contrast use and scanner heterogeneity was considered.

**Results:**

Ninety-five patients met the inclusion criteria. On the basis of the DXA results, we identified 39 normal cases, 46 cases of osteopenia, and 10 cases of osteoporosis. For detecting normal bone, objective CT measurements demonstrated a sensitivity of 88.6% and a specificity of 95.5%. For detecting osteoporosis, the sensitivity was 75.3% and the specificity was 99.7%. For the subjective classification, the level of interobserver agreement was fair (κappa = 0.384). The study included CT scans from different scanner models, with and without contrast enhancement.

**Conclusion:**

Opportunistic CT demonstrated high specificity but only moderate sensitivity for osteoporosis detection. The attenuation thresholds previously validated in populations elsewhere show promise for use in Brazil. However, scanner variability, contrast use, and limited sample size constrain generalizability. Larger, multicenter prospective studies are warranted.

## Abstract (pt)

**Objetivo:**

Avaliar a acurácia diagnóstica da tomografia computadorizada (TC) na detecção de osteoporose em adultos brasileiros, utilizando a absorciometria por dupla energia de raios X (DXA) como padrão de referência.

**Materiais e Métodos:**

Foi realizada uma análise retrospectiva de adultos com mais de 50 anos que se submeteram a exames de TC de tórax, abdome ou coluna lombar e DXA em um intervalo de até 60 dias, entre janeiro de 2012 e dezembro de 2022. A atenuação óssea vertebral em L1 foi quantificada em unidades Hounsfield (UH). Limiares de > 160 UH e < 100 UH foram utilizados para classificar, respectivamente, os ossos normais e a osteoporose. Dois radiologistas musculoesqueléticos realizaram classificações subjetivas de forma independente. Foram calculadas sensibilidade, especificidade, valores preditivos e concordância interobservador (kappa de Cohen). Consideraram-se o uso de contraste e a heterogeneidade dos aparelhos de TC.

**Resultados:**

Noventa e cinco pacientes atenderam aos critérios de inclusão. A DXA identificou 39 casos normais, 46 com osteopenia e 10 com osteoporose. Para a detecção de osso normal, as medidas objetivas da TC apresentaram sensibilidade de 88,6% e especificidade de 95,5%. Para a detecção de osteoporose, a sensibilidade foi de 75,3% e a especificidade, de 99,7%. A concordância kappa para a classificação subjetiva foi de 0,384, indicando concordância razoável. O estudo incluiu exames de TC de diferentes modelos de aparelhos, com e sem contraste.

**Conclusão:**

A TC oportunista demonstrou alta especificidade, mas sensibilidade moderada para a detecção de osteoporose. Os limiares de atenuação validados em populações internacionais mostraram-se promissores para adultos brasileiros. No entanto, a variabilidade entre aparelhos, o uso de contraste e o tamanho limitado da amostra restringem a generalização dos resultados. São necessários estudos prospectivos, multicêntricos e com maior número de pacientes.

## INTRODUCTION

Osteoporosis is a condition that affects women and men worldwide. Among the various complications, fractures of the affected bones directly impact the quality of life of the affected individuals^(1-3)^. These fractures are associated with higher health care costs, as well as with higher rates of morbidity and mortality. Because the incidence of osteoporotic fractures increases with advancing age, measures to diagnose and prevent osteoporosis and its complications have assumed an essential role in the realm of public health^(4)^.

The incidence of osteoporotic fractures varies according to ethnicity and geographical location. The highest rates in men are in Northern Europe and North America. The lowest rates are found in populations of Asian countries and the African continent, as well as in some parts of South America. The female:male ratio among Caucasians is approximately 3-4:1, whereas it is nearly 1:1 or even higher among Asians^(5)^.

During the perimenopausal period, the quantity and quality of bone both decline rapidly, resulting in a dramatic increase in fracture risk in postmenopausal women^(6)^. Although many factors are associated with osteoporotic fractures, routine clinical practice still does not focus on the identification and treatment of women at risk for such fractures. Consequently, osteoporosis is often not diagnosed until a fracture occurs^(2,5-7)^.

Osteoporosis, characterized by reduced bone mineral density (BMD) and microarchitectural deterioration, significantly increases fracture risk and the health care burden worldwide. Dual-energy X-ray absorptiometry (DXA) remains the gold standard for BMD assessment. However, its underutilization in routine practice limits early detection^(8)^.

Introduced into clinical practice in 1987, DXA has largely replaced other methods for assessing BMD. Commonly used for the hip and lumbar spine, DXA can also analyze the calcaneus and distal radius with specialized software^(8)^. Standard spine analysis includes values for each lumbar vertebra from L1 to L4 and a total value for all four, reporting the analyzed area (cm^2^), bone mineral content (g), and BMD (g/cm^2^). In healthy individuals, these values should increase progressively from L1 to L4^(8,9)^. On DXA, each examined area is compared with those of control individuals matched by sex and age (Z-score) and with healthy young individuals at peak bone mass (T-score), as previously described^(8)^. Whereas Z-scores are useful for identifying osteopenia in younger patients, T-scores are used for clinical decision-making in the elderly. Using Z-scores in postmenopausal populations could result in misrepresentation of the incidence of osteoporosis, because patients might appear “normal” compared to their peers despite decreased bone mass and increased fracture risk^(8,9)^.

Before DXA, computed tomography (CT) was employed to diagnose osteoporosis, offering advantages like three-dimensional volumetric analysis and real density measurement, specifically of trabecular bone, which excludes extraneous mineralization^(8)^. However, it has not been confirmed that trabecular bone measurements are more accurate than combined cortical and trabecular determinations. Typically analyzing vertebrae T12 to L3, CT has certain drawbacks^(8-10)^: small changes in site localization or positioning can reduce accuracy; and CT involves a higher radiation dose than does DXA, a significant concern for younger patients requiring serial studies.

Opportunistic screening using CT scans performed for other clinical indications offers a potential solution, allowing vertebral trabecular attenuation to be assessed without additional radiation exposure. Although attenuation thresholds (e.g., > 160 HU for normal bone and < 100 HU for osteoporosis) have been proposed in cohort studies conducted in various countries, the applicability of those thresholds remains underexplored in the Brazilian population^(11-14)^. Therefore, CT can serve as an extremely valuable tool, especially for high-risk patients who may benefit from early intervention before the disease manifests through a fracture^(14)^.

The present study fits into this context: opportunistic bone density analysis by CT for predicting osteoporosis in patients with prior DXA scans, given that osteoporosis remains underdiagnosed and is still an undertreated disease in the orthopedic setting^(15-17)^. Incidental diagnosis is a valuable opportunity to identify low bone mass and initiate treatment, even when the clinical and biological findings are inconclusive^(18,19)^.

Some meta-analyses have shown that population screening is effective in reducing osteoporotic fractures. Therefore, the implementation of screening programs can be useful in preventing bone fractures^(20,21)^.

Emerging automated tools for HU quantification show potential for reproducible, population-level screening strategies. In this context, we aimed to assess the diagnostic performance of CT-derived attenuation values in detecting osteoporosis and to evaluate their potential for opportunistic application in clinical practice for the Brazilian population. The objective of this study was to evaluate the potential of CT as a diagnostic tool for the detection of osteoporosis in patients in Brazil who underwent CT scans for other clinical indications.

## MATERIALS AND METHODS

### Study design and population

This observational, retrospective, cross-sectional study was conducted in accordance with the principles outlined in the Declaration of Helsinki and was approved by the institutional research ethics committee. Because of the retrospective nature of the study, the requirement for informed consent was waived. The study also adhered to Standards for the Reporting of Diagnostic Accuracy Studies guidelines. We reviewed the medical records of patients ≥ 50 years of age who underwent chest, abdominal, or lumbar CT scans that included L1, as well as DXA, between January 2012 and December 2022.

### Inclusion and exclusion criteria

Patients were included in the study if they were ≥ 50 years of age and had undergone CT and DXA scans within a 60-day interval. Patients with vertebral fractures were excluded, as were those with a history of spinal surgery and those with any detectable anatomical variations in the spine.

### CT examination

The proposal for opportunistic osteoporosis screening using CT included the measurement of axial (transverse) trabecular attenuation at L1, together with sagittal reconstruction for vertebral fracture assessment^(15-19)^. The L1 vertebra was selected for measurement because it is typically included in CT scans of the thoracic and lumbar spine, as well as in chest and abdominal CT scans. In cases in which L1 could not be assessed (e.g., due to artifact-induced changes or only partial inclusion in the examination), an adjacent level such as T12 or L2 was measured instead^(17)^.

### CT acquisition

For image acquisition and attenuation analysis, we employed multidetector CT scanners with 40, 80, or 320 detector rows, following standard institutional protocols. We measured trabecular attenuation at L1 in a standardized oval region of interest (ROI) of 100-300 mm^2^ on axial slices, avoiding cortical bone and vascular structures. Values > 160 HU were classified as normal, whereas those < 100 HU were considered indicative of osteoporosis^(17)^. Unenhanced and contrast-enhanced CT scans were included. The specific models utilized were the Biograph mCT (Siemens Healthcare, Erlangen, Germany), Aquilion Prime (Canon Medical Systems, Tochigi, Japan), and Aquilion ONE (Canon Medical Systems). The scanning parameters adhered to institutional protocols, with scans being acquired when the patient was in the supine position and during end-inspiration. Contrast material was administered intravenously as needed. The reconstructed slice thickness was set at 1 mm, with a tube voltage of 80-120 kVp and an automatically modulated tube current of 10-440 mA. The CT scans were acquired in outpatients, inpatients, and emergency room patients; in the last two cases, the scans were always acquired at admission.

### DXA

All of the DXA examinations performed at our institution comply with the regulations established by Brazilian national organizations, and all of the equipment employed undergoes regular maintenance. The DXA analysis was performed with a fan-beam densitometer (iDXA; GE Healthcare, Madison, WI, USA). We categorized bone quality on the basis of the T-scores, applying the World Health Organization criteria^(22)^: normal (≥ -1), osteopenia (-1 to -2.5), and osteoporosis (< -2.5). All DXA examinations were conducted in outpatients.

### Image analysis

**Figure 1:**
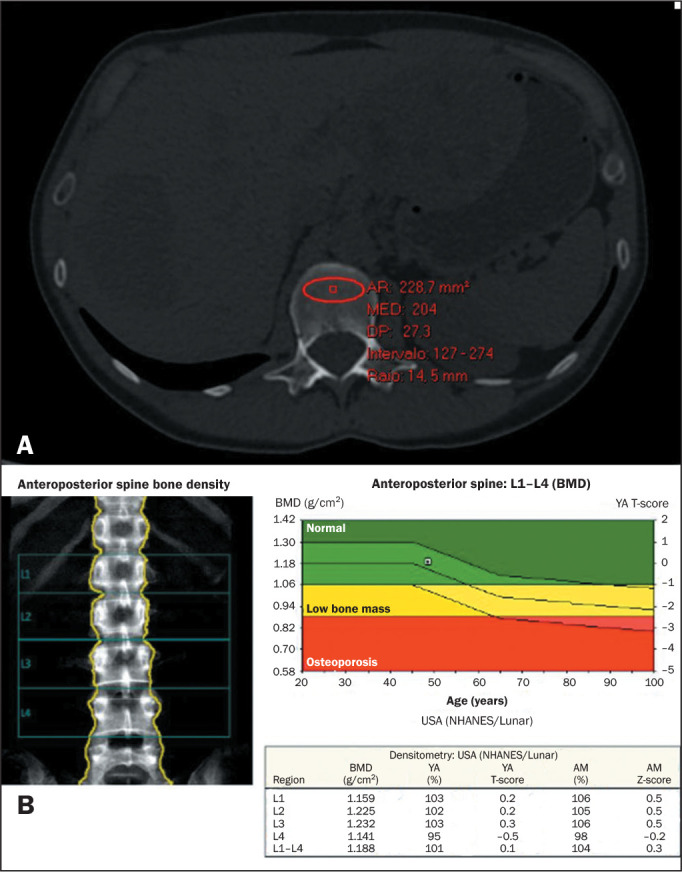
A: Abdominal CT scan with bone window settings, performed on a 62-year-old woman to evaluate abdominal pain and distention in an urgent context. The ROI for trabecular attenuation measurement was placed at the L1 level in this axial CT slice. The mean trabecular attenuation was 204 HU. B: DXA screening performed on the same patient 32 days before the CT examination. The DXA image shows a normal result (normal value ≥ 1), with a T-score of 0.1 at L1-L4.

AR, area; MED, mean; DP, standard deviation; Intervalo, range; Raio, ROI radius; NHANES, National Health and Nutrition Examination Survey; YA, young adult; AM, age-matched.

**Figure 2:**
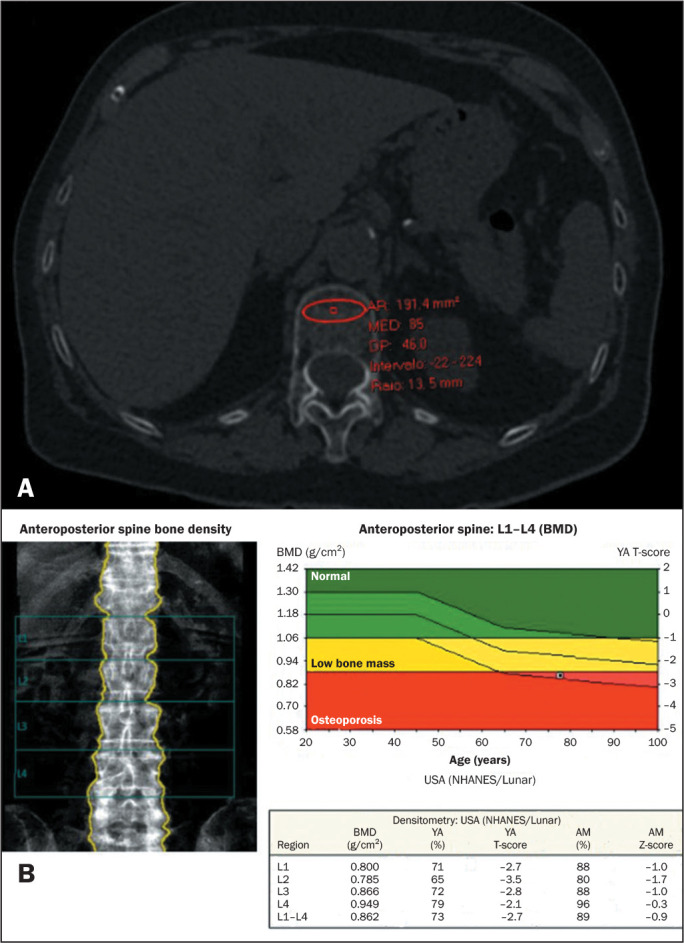
A: Abdominal CT of a 69-year-old woman, performed to investigate renal calculi in an emergency room setting. The ROI for trabecular attenuation measurement was placed at the L1 level in this axial CT slice. The trabecular attenuation was low (85 HU). B: DXA screening was performed on the same patient 10 days before the CT. The DXA image shows osteoporosis, with a T-score of -2.7 at L1-L4.

AR, area; MED, mean; DP, standard deviation; Intervalo, range; Raio, ROI radius; NHANES, National Health and Nutrition Examination Survey; YA, young adult; AM, age-matched.

Each CT examination included the L1 trabecular space. Vertebral assessment on CT was performed by the evaluators on a standard picture archiving and communication system workstation; the images were viewed with the bone window settings routinely employed at our institution. A single ROI measuring 100-300 mm^2^ was placed within the trabecular bone of L1 in the transverse (axial) series to assess mean attenuation values^(20-22)^. No angulation was required. In all cases, structures that could alter the ROI value, such as artifacts or vessels, were avoided (Figures 1 and 2).

Two musculoskeletal radiologists, with three and seven years of experience, respectively, performed independent subjective evaluations of L1 on sagittal reconstructions. Both were blinded to the DXA results and all clinical data. Interobserver agreement was assessed by using weighted kappa statistics. The analysis was conducted in two stages. In the first stage, images obtained in the bone window with sagittal reconstruction were employed in order to classify the lumbosacral spine, subjectively, as normal, osteopenic, or osteoporotic. In the second stage, the more experienced radiologist quantified bone density using the methodology outlined by Graffy et al.^(22)^ and other authors^(23,24)^.

For BMD assessment, DXA was considered the gold standard. On the basis of the DXA results, bone segments were classified as normal, osteopenic, or osteoporotic, according to the World Health Organization criteria for categorizing T-scores^(22,25,26)^.

### Statistical analysis

Diagnostic metrics, including sensitivity, specificity, positive predictive value (PPV), and negative predictive value (NPV) were calculated to assess the performance of CT compared to DXA. Agreement in attenuation was evaluated using Bland-Altman plots, with 95% limits of agreement for continuous outcomes. The osteoporosis classifications, subjectively evaluated by two musculoskeletal radiologists, were compared by using contingency tables, and interobserver agreement was quantified with weighted kappa coefficients. Kappa values were interpreted according to the categories established by Landis and Koch^(27)^: < 0.00, poor; 0.00-0.20, slight; 0.21-0.40, fair; 0.41-0.60, moderate; 0.61-0.80, substantial; and 0.81-1.00, almost perfect. We considered predictive values in the context of disease prevalence, noting that the high prevalence of osteopenia/osteoporosis in this cohort could overestimate PPVs in comparison with general screening populations. Statistical analyses were performed with the IBM SPSS Statistics software package, version 22.0 for Windows (IBM Corp., Armonk, NY, USA). A senior statistician with over ten years of experience in biomedical research tabulated the data in Microsoft Excel. Odds ratios and 95% confidence intervals were calculated, and (two-sided) values of *p* < 0.05 were considered statistically significant.

## RESULTS

### Study population

**Figure 3:**
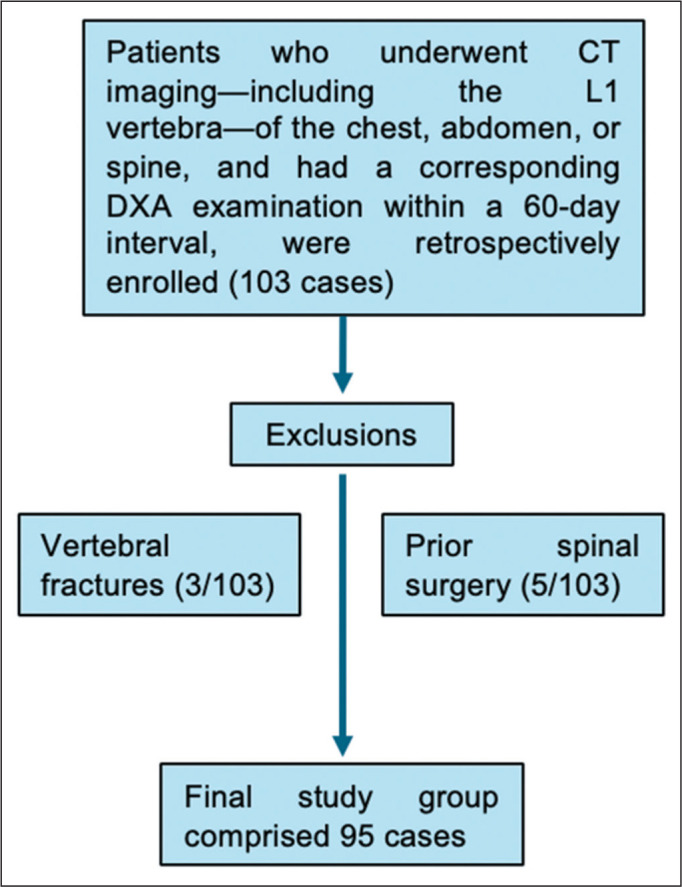
Flow chart of patient inclusion and exclusion. We included a total of 95 patients, all over 50 years of age, who underwent both CT and DXA scans within a 60-day interval. From the initial sample of 103 patients, eight were excluded: three because they had vertebral fractures; and five because they had a history of spinal surgery.

Of the 103 patients initially identified (98 women and 5 men), 95 had undergone CT and DXA scans within a 60-day interval and met the other inclusion criteria. On the basis of the DXA results, we classified 39 patients as normal, 46 as having osteopenia, and 10 as having osteoporosis. As illustrated in Figure 3, patients with vertebral fractures were excluded, as were those with a history of surgical intervention and those with variations in vertebral morphology^(15-17)^. All patients were subjectively and objectively assessed by the most experienced radiologist, who first evaluated the bone density and then measured the attenuation in the lumbar spine.

### Objective evaluation: normal bone versus osteopenic/osteoporotic bone

**Figure 4:**
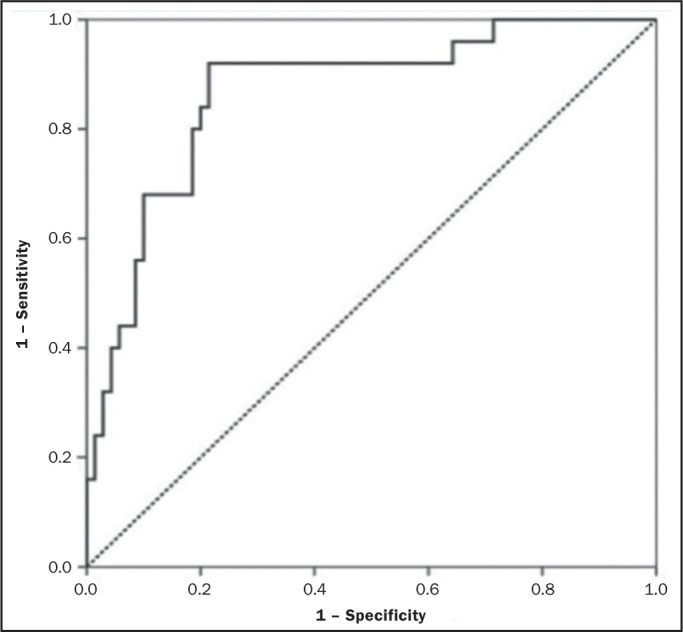
ROC curve representing the diagnostic performance of CT for detecting normal bone versus osteopenic or osteoporotic bone. The area under the curve reflects the overall accuracy of the test in distinguishing between normal and abnormal bone density.

In the objective evaluation aimed at distinguishing normal bone from bone that was osteopenic or osteoporotic, the diagnostic performance of CT was assessed by using standard metrics. The prevalence of osteopenia/osteoporosis in the sample was 82.2%. The sensitivity of CT was 88.6%, indicating a high ability to correctly identify patients with reduced bone density, and its specificity was 95.5%, reflecting an excellent capacity to correctly identify individuals with normal bone. The PPV of CT was 98.2%, suggesting that most patients classified as having osteopenia or osteoporosis on the basis of CT images truly had the condition, and its NPV was 76.1%, indicating a moderate ability to rule out bone abnormalities when CT findings are normal. These results, as depicted in Figure 4, highlight the overall accuracy of CT for the detection of altered bone density, particularly for confirming a diagnosis of osteopenia or osteoporosis.

### Objective evaluation: osteoporotic bone versus normal/osteopenic bone

**Figure 5:**
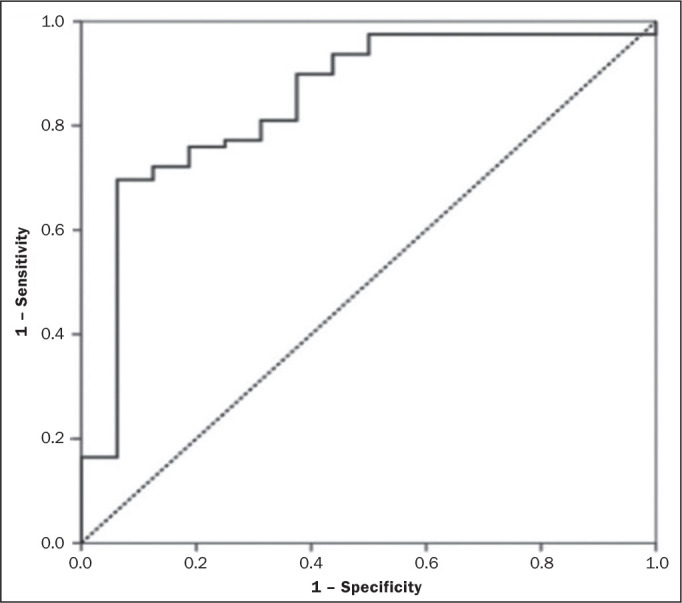
ROC curve representing the diagnostic performance of CT for detecting normal or osteopenic bone versus osteoporotic bone. The area under the curve reflects the overall accuracy of the test in distinguishing between normal and abnormal bone density.

In the objective evaluation aimed at distinguishing osteoporotic bone from normal or osteopenic bone, CT demonstrated good diagnostic performance for confirming the presence of osteoporosis. The sensitivity was 75.3%, indicating a moderate ability to identify affected individuals, whereas the specificity was 99.7%, showing excellent performance in ruling out the disease in unaffected individuals. The PPV was 97.5%, and the NPV was 95.8%, underscoring the accuracy of CT in confirming the diagnosis of osteoporosis. However, because of its lower sensitivity, it may be less suitable for screening purposes. The detailed results are illustrated in Figure 5.

### Kappa statistic: qualitative assessment of interobserver agreement

The interobserver agreement for categorical data was assessed by calculating the kappa statistic, as described by Landis and Koch^(27)^. The kappa value for subjective CT analysis between the two radiologists was 0.384, indicating fair agreement. Discrepancies were most common in relation to the distinction between osteopenic bone and normal bone, as shown in Table 1.

**Table 1:** Interobserver agreement for the classification of subjects as normal, with osteopenia, and with osteoporosis.

		Observer 2			
		Normal	Osteopenia	Osteoporosis	Total
Observer 1	Normal	**31**	5	0	36
	Osteopenia	22	**21**	4	47
	Osteoporosis	2	3	**7**	12
	Total	55	29	11	95

Overall, the two observers were in agreement in 59 (62.1%) of the 95 cases evaluated. Cohen’s kappa coefficient was 0.379 (95% CI: 0.231-0.537; *p* < 0.05), indicating fair agreement. Most disagreements were in relation to the distinction between normal bone and osteopenic bone.

## DISCUSSION

In this retrospective analysis, we evaluated the utility of opportunistic CT screening for assessing BMD using previously validated attenuation thresholds. An objective analysis demonstrated that such screening has high specificity and sensitivity (95.5% and 88.6%, respectively) in identifying normal bone density, supporting the use of > 160 HU as a reliable threshold for exclusion of osteoporosis. In contrast, when evaluating bones with osteoporosis, CT showed excellent (99.7%) specificity but only moderate (75.3%) sensitivity, underscoring its value in confirming-but not excluding-disease. In addition, our findings indicate that the subjective assessment of osteoporosis shows poor reliability, with low interobserver agreement for the classification of bone as normal, osteopenic, or osteoporotic. The kappa value for agreement between the two radiologists was 0.384, indicating only fair concordance. This suggests that subjective evaluation of bone conditions may not provide consistent or reliable results, highlighting the need for approaches to osteoporosis assessment that are more objective and standardized.

Osteoporosis remains a highly prevalent and widely underdiagnosed condition, associated with substantial health care costs, morbidity, and mortality^(1-3,5,16,20,21)^. Opportunistic CT screening has emerged as a pragmatic approach, particularly in populations with limited access to DXA, offering a cost-effective, radiation-neutral method for expanding detection rates^(11-14)^. Although cohort studies conducted in Europe, such as that of Merlijn et al.^(19)^, have shown that population-wide screening strategies can reduce fracture incidence^(20,21)^, few data are available for populations in Latin America.

Using the methodology proposed by Graffy et al.^(22-24)^, our study supports the applicability of the > 160 HU and < 100 HU thresholds in Brazil. These values were originally validated in populations in southern Europe and the United States^(17,28)^; our findings provide preliminary evidence that they are also effective in Latin America. Nevertheless, as suggested in previous studies^(22)^, lower thresholds (e.g., 90 HU) may offer improved sensitivity in identifying osteoporosis and should be further evaluated in Brazil.

The present study demonstrated moderate interobserver agreement for the subjective assessment of osteoporosis. This finding is consistent with those of other studies that have evaluated the subjective assessment of bone conditions. For instance, Vigers et al.^(29)^ investigated interobserver agreement in radiographic assessments of wrist osteopenia, analyzing subjective evaluations in comparison with DXA results. Those authors reported limited agreement, with a 51% concordance rate, indicating that subjective radiographic assessments may not reliably identify osteopenia without the use of additional imaging modalities. Similarly, Mulugeta et al.^(30)^ found that radiographs of the appendicular skeleton had low sensitivity for detecting osteopenia in children, with interobserver agreement for severe osteopenia ranging from 0% to 25%, further highlighting the challenges of subjective assessment in the absence of fractures. These studies underscore the inherent variability in subjective assessments of osteoporosis and osteopenia, indicating that factors such as observer experience and the lack of standardized criteria contribute to inconsistent results. As demonstrated in our study, these limitations support the need for approaches to the diagnosis of osteoporosis that are more objective and standardized.

In the objective analysis performed by the most experienced radiologist in our study, opportunistic CT screening showed high (88.6%) sensitivity and high (95.5%) specificity in detecting normal bones, with a PPV of 88.6% and a NPV of 95.5%, demonstrating good accuracy in ruling out bone alterations. The > 160 HU threshold proved to be reliable in excluding trabecular bone alterations in L1. These data indicate that opportunistic CT screening shows promise for use in the population of Brazil, as observed in the United States by Boutin and Lenchik^(17)^.

In the present study, the radiologist found that opportunistic CT screening showed good specificity (99.7%) but moderate sensitivity (75.3%) in identifying bones with osteoporosis, with a PPV of 97.5% and a NPV of 98.5%. The < 100 HU threshold showed promise in confirming the presence of osteoporotic disease in the vertebra evaluated. However, caution should be exercised regarding screening programs for assessing these bones in the population of Brazil. Some studies, such as that conducted Graffy et al.^(22)^, used a cutoff of ≤ 90 HU for detecting fractures in L1. That threshold might present greater accuracy in detecting osteoporotic bone in the Brazilian population. Therefore, the values used for opportunistic screening of osteoporosis and for detecting vertebrae with normal BMD also proved promising for the evaluated population (of Brazil in this case).

The present study presents several positive aspects, particularly the use of DXA as the gold standard for bone assessment, providing a reliable comparison for evaluating the potential of CT for the detection of osteoporosis. The focus on the Brazilian population, which remains underrepresented in the current literature, offers a valuable contribution to the field. To simulate real-life clinical practice, we included unenhanced and contrast-enhanced CT scans, both of which are common in routine clinical settings, allowing a more representative evaluation of the performance of CT in diagnosing osteoporosis. This real-world approach strengthens the relevance of our study for potential implementation in clinical practice, given that it mirrors the diversity of conditions and procedures encountered in actual diagnostic scenarios.

Several positive findings emerged from the present study, including the confirmation that CT-derived attenuation thresholds exhibit high specificity but only moderate sensitivity for osteoporosis detection. These findings suggest that CT can effectively rule out bone alterations but may be less reliable for detecting all cases of osteoporosis, especially in a heterogeneous clinical setting. However, certain important limitations must be acknowledged:

• Variability in acquisition protocols-Scans were performed over an 11-year period, utilizing unenhanced and contrast-enhanced techniques. The lack of control for intravenous contrast, which is a factor known to affect bone marrow attenuation, likely reduced the sensitivity of CT and limited the comparability of results. Despite this, the inclusion of contrast-enhanced examinations simulates the variability encountered in real-world clinical practice, in which contrast use is often determined by clinical indications.

• Scanner heterogeneity-Imaging was performed on scanners with 40-320 detector rows, without any inter-scanner calibration. This technical variability could have influenced attenuation measurements, affecting the consistency of results. A protocol that was more standardized across scanner models would have strengthened the reliability of the findings.

• Limited sample size-With only 10 patients having confirmed osteoporosis, the sample size was small, which reduces statistical power and limits the external validity of the study. In addition, the high prevalence of osteopenia/osteoporosis in the cohort is not representative of a typical screening population, potentially leading to overestimation of the PPVs.

• Use of fixed attenuation thresholds-Although the use of fixed attenuation thresholds (e.g., > 160 HU for normal bone and < 100 HU for osteoporosis) is practical, it may not account for population-specific variations in bone density. The lack of a ROC curve analysis to determine the optimal thresholds means that the findings might not be generalizable across diverse populations.

• Subjective interpretation-The moderate interobserver agreement (κ = 0.384) for subjective classification of bone conditions suggests that subjective interpretation alone may not be sufficient for accurate diagnosis. This highlights the need for standardized training or the development of automated tools to improve reproducibility and reduce observer bias.

• Retrospective design-Given that this was a retrospective study, the observational nature introduced inherent limitations, such as selection bias and a lack of protocol standardization, which may have affected the consistency of the results.

• Gender imbalance-The study population consisted predominantly of women (90 women vs. 5 men). Given that BMD differs between sexes, this gender imbalance may limit the generalizability of the findings, which are likely more representative of the female population.

• Selection bias-We included only patients over 50 years of age who underwent CT and DXA, which increases the likelihood of pre-existing osteopenia or osteoporosis. This selection bias makes our findings inapplicable to younger, asymptomatic populations or to those undergoing CT for non-osteoporotic indications.

Despite these limitations, our study provides valuable insights, particularly for regions in which access to DXA is limited. The use of CT-based screening protocols could expand diagnostic capacity, provided that efforts are made to standardize protocols and further validate the findings. These results underscore the need for larger, prospective studies that stratify data by scanner type, contrast use, and patient characteristics, as well as having the aim of establishing population-specific attenuation thresholds through rigorous ROC curve analyses.

## CONCLUSION

The CT-derived trabecular attenuation thresholds evaluated here demonstrated overall excellent specificity and good diagnostic performance for confirming normal bone status and identifying osteoporosis, supporting their potential role in opportunistic screening. Our findings suggest that previously established attenuation cutoffs (> 160 HU to exclude and < 100 HU to confirm osteoporosis) may be applicable to adults in Brazil, a population underrepresented in the literature on diagnostic imaging for osteoporosis. In resource-limited settings, such methods offer a pragmatic alternative to expand diagnostic access without additional cost or radiation exposure. However, limitations-including the small sample size, scanner/protocol heterogeneity, and the unmeasured effect of contrast enhancement-limit the immediate clinical applicability of our findings. Further multicenter, prospective studies with stratified analyses and population-specific threshold optimization are essential in order to validate this approach and support its broader implementation in routine practice.

## Data Availability

Data sets related to this study will be available upon request to the corresponding author.
